# Ultrafast carbothermal reduction of silica to silicon using a CO_2_ laser beam

**DOI:** 10.1038/s41598-020-78562-1

**Published:** 2020-12-10

**Authors:** Seok-Ho Maeng, Hakju Lee, Min Soo Park, Suhyun Park, Jaeki Jeong, Seongbeom Kim

**Affiliations:** 1 Department of Mechanical Design Engineering, Kangwon National University, Samcheok-si, 25913 Republic of Korea; 2 Laboratory of Photomolecular Science, Institute of Chemical Sciences Engineering, Ecole Polytechnique Federale de Lausanne (EPFL), 1015 Lausanne, Switzerland

**Keywords:** Materials for devices, Process chemistry

## Abstract

We report the extraction of silicon via a carbothermal reduction process using a CO_2_ laser beam as a heat source. The surface of a mixture of silica and carbon black powder became brown after laser beam irradiation for a few tens of seconds, and clear peaks of crystalline silicon were observed by Raman shift measurements, confirming the successful carbothermal reduction of silica. The influence of process parameters, including the laser beam intensity, radiation time, nitrogen gas flow in a reaction chamber, and the molar ratios of silica/carbon black of the mixture, on the carbothermal reduction process is explained in detail.

## Introduction

Silicon is one of the most essential materials for electronic devices. Integrated circuit chips fabricated on a silicon wafer are currently used in almost all devices, making our daily lives more convenient than ever before. Moreover, the demand for silicon wafers is increasing due to the photovoltaic industry in which crystalline silicon solar cells play a major role, and the production cost of silicon wafers is critical in the commercialization of silicon solar cells. Indeed, advances in silicon technologies have revolutionized modern society in terms of the development of ubiquitous electronics and energy technology and associated industries.

Silicon is an abundant element in Earth’s crust; however, it exists as silicon oxides, mostly as silica. It requires considerable time and energy to produce pure silicon from silica by reduction because of the high bonding energy between silicon and oxygen atoms^1^. In industry, carbothermal reduction using an electric arc furnace is adopted to produce pure silicon from silica because this system is considered to be the most economical route in large-scale production^2–4^. Carbothermal reduction using an arc furnace has been thoroughly investigated to date^5–8^; however, the traditional carbothermal reduction process requires large-scale facilities to achieve high thermal efficiency during the high-temperature process and requires a stabilization process for normal operation after process initiation^3^. Although carbothermal reduction using an arc furnace has contributed to the large-scale production of metallurgical silicon, it does not seem to be a general solution for obtaining silicon through the reduction of silica, especially when the reduction process should occur in a specific region or on a small scale.

A few novel approaches for the reduction of silica have been reported to date. Successful electrochemical reduction of silica to silicon has been reported^9–11^. This electrochemical reduction process enabled the process temperature to be less than 1000 °C and enabled the reduction reaction to take place at a contact point with an electrode. Other approaches, such as carbothermal reduction of silica using concentrated solar energy^12^ and reduction of silica from rice husks, have been reported^13,14^. Although these attempts incorporate additional metals or chemicals other than silica and carbon, they involve meaningful routes for producing silicon without using traditional silica reduction processes.

In this paper, for the first time, we report evidence of reduced silicon from silica by ultrafast carbothermal reduction using a CO_2_ laser beam. A CO_2_ laser is considered an excellent energy source in terms of heat flux; however, no report has yet studied the carbothermal reduction of silica using a CO_2_ laser beam. We observed that carbothermal reduction took place within a few seconds of the laser beam illuminating the silica/carbon mixture. The intrinsic peaks of crystalline silicon were detected from the silica/carbon mixture after laser beam irradiation by Raman spectroscopy. Our experimental results confirmed that the intensity of the laser beam and the N_2_ gas flow into the reaction chamber largely influenced the carbothermal reduction process.

## Experimental details

**Figure 1:**
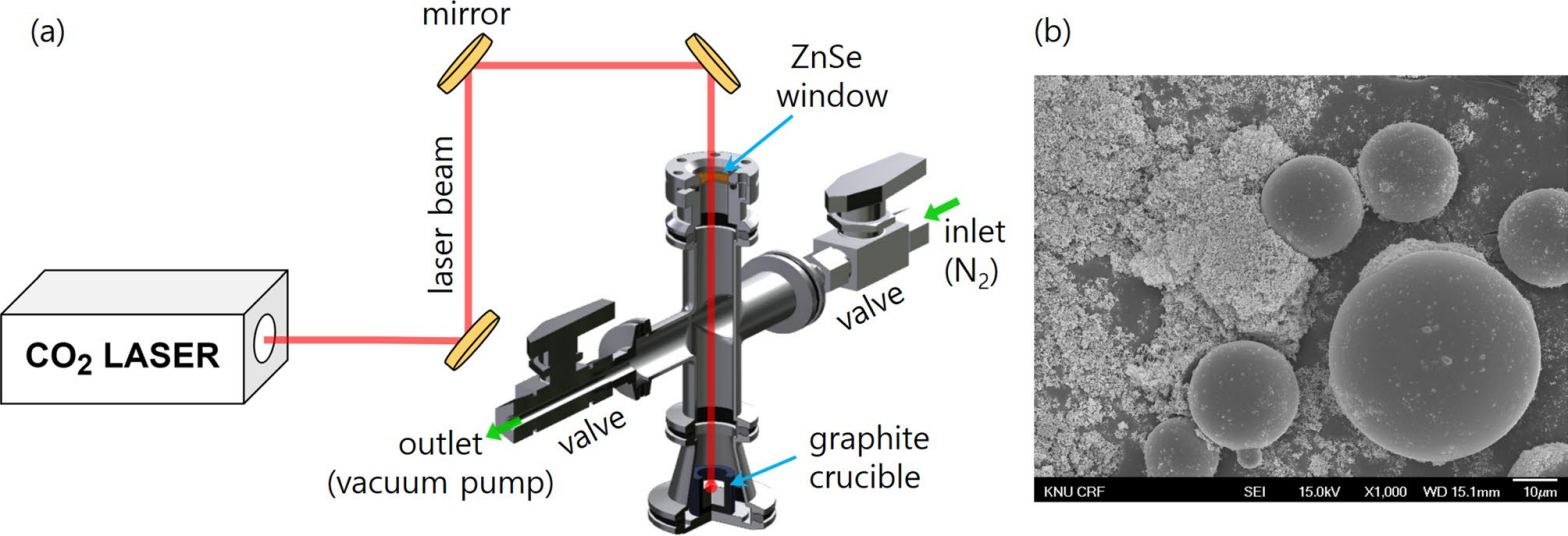
(**a**) A schematic of the experimental setup and (**b**) a SEM image of the pristine mixture.

A schematic of the experiment is depicted in Fig. 1(a). A CO_2_ laser beam with a 10.6 µm wavelength (continuous wave, maximum power 120 W, Model: SR 10i, Rofin-Sinar UK Ltd.) was used as an energy source. A raw laser beam with a 6 ± 0.5 mm diameter defined by 1/e^2^ intensity from a Gaussian profile was used without any focusing lens. A simple reactor was designed to isolate chemical reactions upon a laser beam from ambient air. The reactor had a window made of ZnSe for the laser beam entrance and two gas flow lines for the N_2_ gas inlet and outlet. A rotary vacuum pump was connected to the outlet to keep fumes and gases draining to the outlet for all the experiments. A mixture of silica (0–0.05 mm Cat. No. Si5210, Daihan agent) and carbon black (VULCAN XC72R, Cabot Corp.) powder was loaded in a 13 mm inner diameter graphite crucible. The loading weight of the mixture was 200 mg, and the molar ratio of silica to carbon was 1:4. The mixture was compressed into the crucible with a 5 kg force using a steel block and a customized tool to make the powder surface even and compact. The crucible with the mixture was heated on a hot plate at 100 °C for an hour to remove residual moisture in the sample. A scanning electron microscopy (SEM) image of the pristine mixture is shown in Fig. 1(b) and shows that silica beads tens of micrometers in diameter are surrounded by carbon black powder. The reactor was vacuumed before the laser beam radiation, and N_2_ gas flow was regulated using a mass flow controller (MFC) during the process to control the atmosphere of the reactor and to protect the surface of the ZnSe window from contamination due to vaporized substances. The N_2_ gas flow rate was 5000 standard cubic centimeters per minute (sccm), and the pressure inside the reactor was approximately 100 Torr.

## Results and discussion

**Figure 2:**
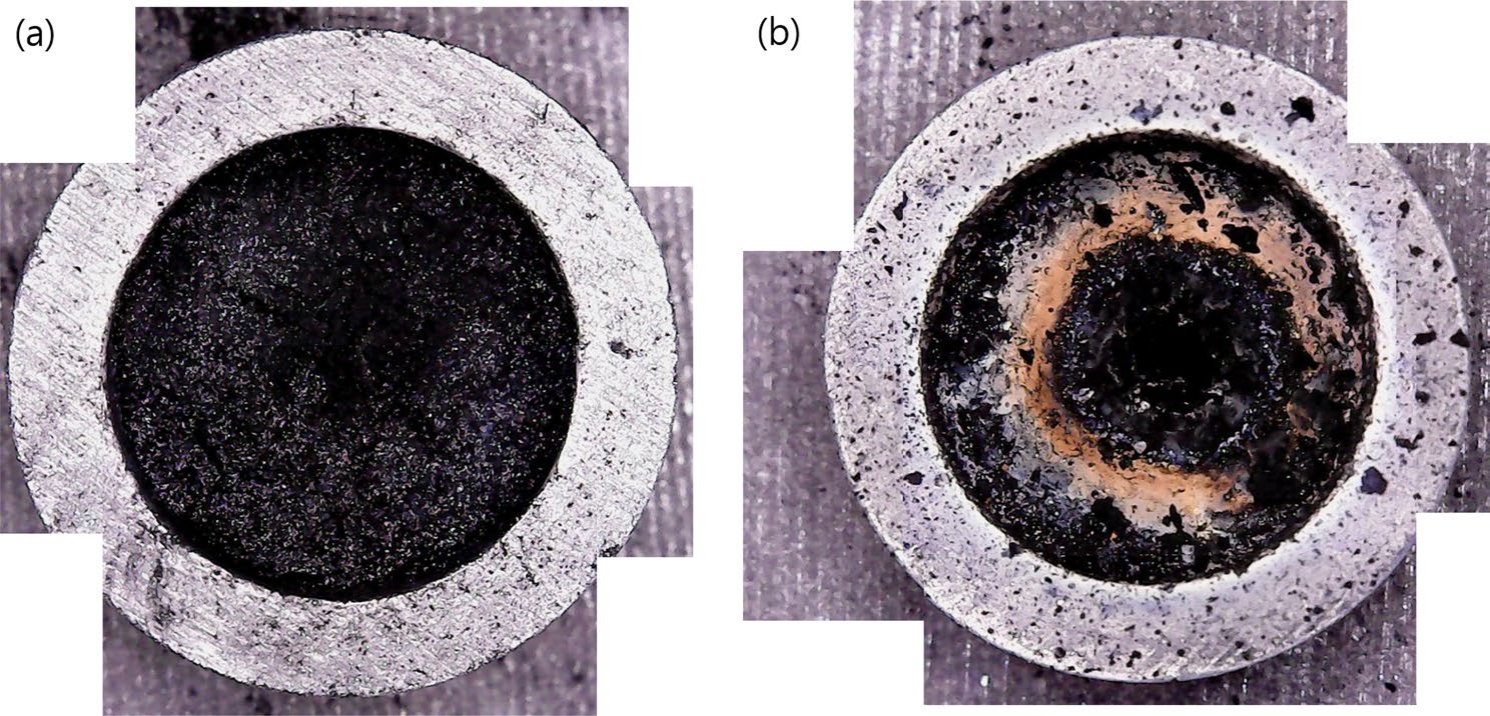
Digital images of the samples contained in a graphite crucible after (**a**) 25 W and (**b**) 50 W laser beam irradiation.

The laser beam radiation caused instantaneous heating of the surface of the mixture, which induced radical phase changes and chemical reactions within a local area before reaching the equilibrium status of the global system. Figure 2 shows top-view pictures of the samples after laser beam irradiation. Significant color changes are observable for the sample subjected to 50 W irradiation (Fig. 2b) in comparison with the sample subjected to 25 W irradiation (Fig. 2a). In both samples, a crater was formed at the irradiated spot. The instantaneous heating by the laser beam melted the silica beads, and the melted silica beads were condensed. At the same time, the chemical reaction generated fumes and gases consuming the powder mostly on the irradiated spot. The size and depth of the crater increased as the laser beam radiation power and time increased. The area around the crater of the 50 W irradiated sample became brown, but there were no changes in color of the 25 W irradiated sample except in the shallow depth of the crater. The brown area was a thin layer of fine powder. The thickness of the brown layer was irregular but estimated to be from a few hundred nanometers to a few tens of micrometers.

**Figure 3:**
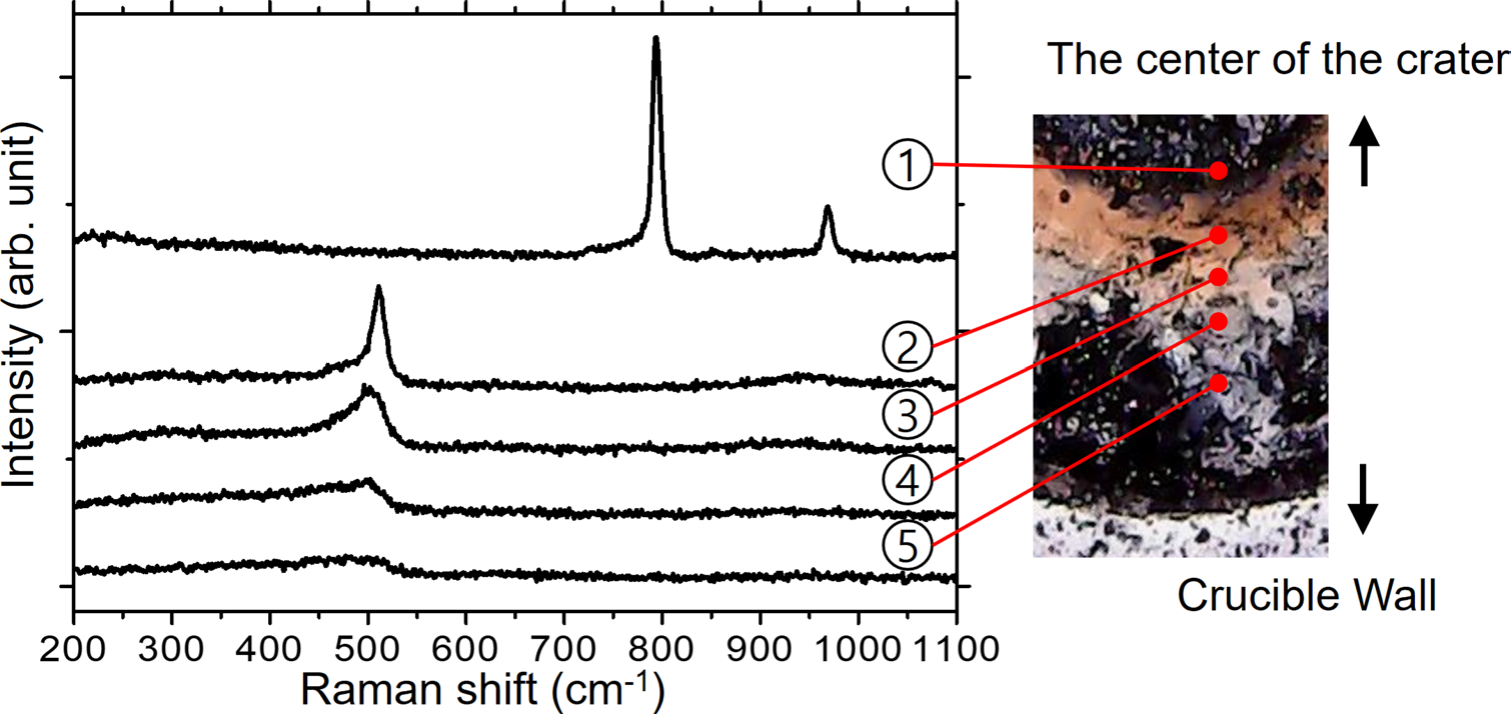
Raman spectra of the sample after 50 W laser beam irradiation.

A small piece of brown powder was investigated using a micro-Raman spectrometer. The Raman spectra were collected with a 532 nm wavelength, 2 mW power laser beam. The Raman spectra in Fig. 3 show 5 different signals from each area identified in the sample picture on the right side of the figure. Intrinsic peaks of cubic SiC at 794 and 970 cm^−1^ are seen from the spectrum of point 1, which is contiguous to the crater^15^. It has been widely reported that SiC plays an essential role in the carbothermal reduction of silica as an intermediate compound^2,16,17^. The SiC peaks disappeared and a crystalline silicon peak was detected at point 2, where the brown color was observed, implying that silicon has been successfully reduced in this area. The silicon peak was located at 511 cm^−1^, which was shifted from the peak of stress-free, bulk crystalline silicon at 521 cm^−1^. We propose that the shift arises from small silicon crystallites, grain boundaries and thermal stress during the process^18–20^. It was also observed that the broad amorphous silicon peak at approximately 480 cm^−1^ contributes to the shoulder of the peak. The intensity of the crystalline silicon peak was reduced as the measured point shifted outwards, as shown in the spectra of points 3, 4 and 5, which is consistent with the changes in the color of the sample. The tendencies based on the Raman spectra, including clear peaks of SiC and Si according to the different areas of the sample, may imply that there is a favorable reaction zone or conditions for carbothermal reduction.

The maximum intensity of the laser beam was estimated to be ~ 4.29 × 10^6^ W/m^2^ when the total power was 50 W, provided that the laser beam followed a Gaussian profile, and this intensity was considered the critical intensity for carbothermal reduction in our experimental setup. A laser beam intensity of ~ 2.15 × 10^6^ W/m^2^ at 25 W total power condition was sufficient to melt the silica beads; however, it was not sufficient to initiate the reduction reaction. Therefore, it is reasonable to assume that the threshold intensity for the carbothermal reduction reaction using a CO_2_ laser beam is close to 4.29 × 10^6^ W/m^2^. Given that the laser beam intensity followed a Gaussian profile, the temperature decreased as the measurement point shifted further from the center of the irradiated area. The temperature at the center of the irradiated spot was measured using an infrared pyrometer during laser beam radiation. The maximum temperature reached 1780 and 2100 °C for 25 and 50 W total laser power conditions, respectively. The temperature reached its maximum as soon as laser beam illumination began, and this is considered the most beneficial characteristic when the laser beam is used as a heat source at a local area. Note that the laser beam radiation time was 10 s. While the in situ diagnosis of the reaction mechanism within the chamber will not be discussed due to a lack of proper technique in the present paper, the carbothermal reduction reaction seemed to take place on the order of seconds. We believe that this is the fastest carbothermal reduction reaction of silica to silicon ever reported.

A few process parameters were controlled to investigate their effects on the reduction reaction. When the experiment was performed under vacuum conditions without N_2_ gas flow, silicon, which was shown as the brown area, was not observed at any laser power, radiation time or molar ratio of silica and carbon. If the temperature of the local area under the laser beam radiation was the necessary and sufficient factor, a signal of the silicon from the sample would have been observed under vacuum condition. This provides insight into the reduction mechanism at the confined area in our system, suggesting that mass transport of the gas phase is highly involved in the reaction^21^. It is well understood that silicon is not produced by the direct reduction of silica by carbon. Several reactions take place during the carbothermal reduction of silica to silicon as follows^2,3^

1 $$ {\text{SiO}}_{{2}} \left( {\text{s}} \right) + {\text{C}}\left( {\text{s}} \right) = {\text{SiO}}\left( {\text{g}} \right) + {\text{CO}}\left( {\text{g}} \right) $$

2 $$ {\text{2SiO}}_{{2}} \left( {\text{s}} \right) + {\text{SiC}}\left( {\text{s}} \right) = {\text{3SiO}}\left( {\text{g}} \right) + {\text{CO}}\left( {\text{g}} \right) $$

3 $$ {\text{SiO}}_{{2}} \left( {\text{s}} \right) + {\text{Si}}\left( {\text{l}} \right) = {\text{2SiO}}\left( {\text{g}} \right) $$

4 $$ {\text{SiO}}\left( {\text{g}} \right) + {\text{2C}}\left( {\text{s}} \right) = {\text{SiC}}\left( {\text{s}} \right) + {\text{CO}}\left( {\text{g}} \right) $$

5 $$ {\text{SiO}}\left( {\text{g}} \right) + {\text{SiC}}\left( {\text{s}} \right) = {\text{2Si}}\left( {\text{l}} \right) + {\text{CO}}\left( {\text{g}} \right) $$

SiC is inevitably involved in Si production as an intermediate. The production of Si is determinative of the chemical reaction (5). If these chemical reactions occur in our system, it is important to control the partial pressure of gaseous SiO and CO inside the reactor^2^. Under vacuum conditions without a N_2_ gas flow in our experiments, it was assumed that not only CO but also gaseous SiO were drained to the outlet without undergoing chemical reaction (5). In contrast, under nonvacuum conditions, silicon was easily produced when N_2_ gas was introduced to the reactor. We expect that the N_2_ gas flow might have influenced gaseous SiO by swirling around the irradiated area. Then, Si could be produced around the favorable temperature area by a chemical reaction (5), as shown by the brown ring in Fig. 2(b). It is expected that silicon is present in the brown ring because the gases are generated actively in the radiation area.

During traditional carbothermal reduction, the ratio of silica and carbon is important for the production of silicon in terms of the process temperature of the crucible and the production yield^3,22,23^. We prepared mixture samples with molar ratios of silica to carbon black varying from 1:1 to 1:10 and conducted carbothermal reduction using a laser beam. Interestingly, silicon was successfully produced from all the samples. Radical and localized heating upon laser beam irradiation might account for the results; otherwise, the vapor pressures of the generated gases according to a thermally equilibrated state might be influenced by the molar ratio of silica to carbon in the typical carbothermal reduction system.

**Figure 4:**
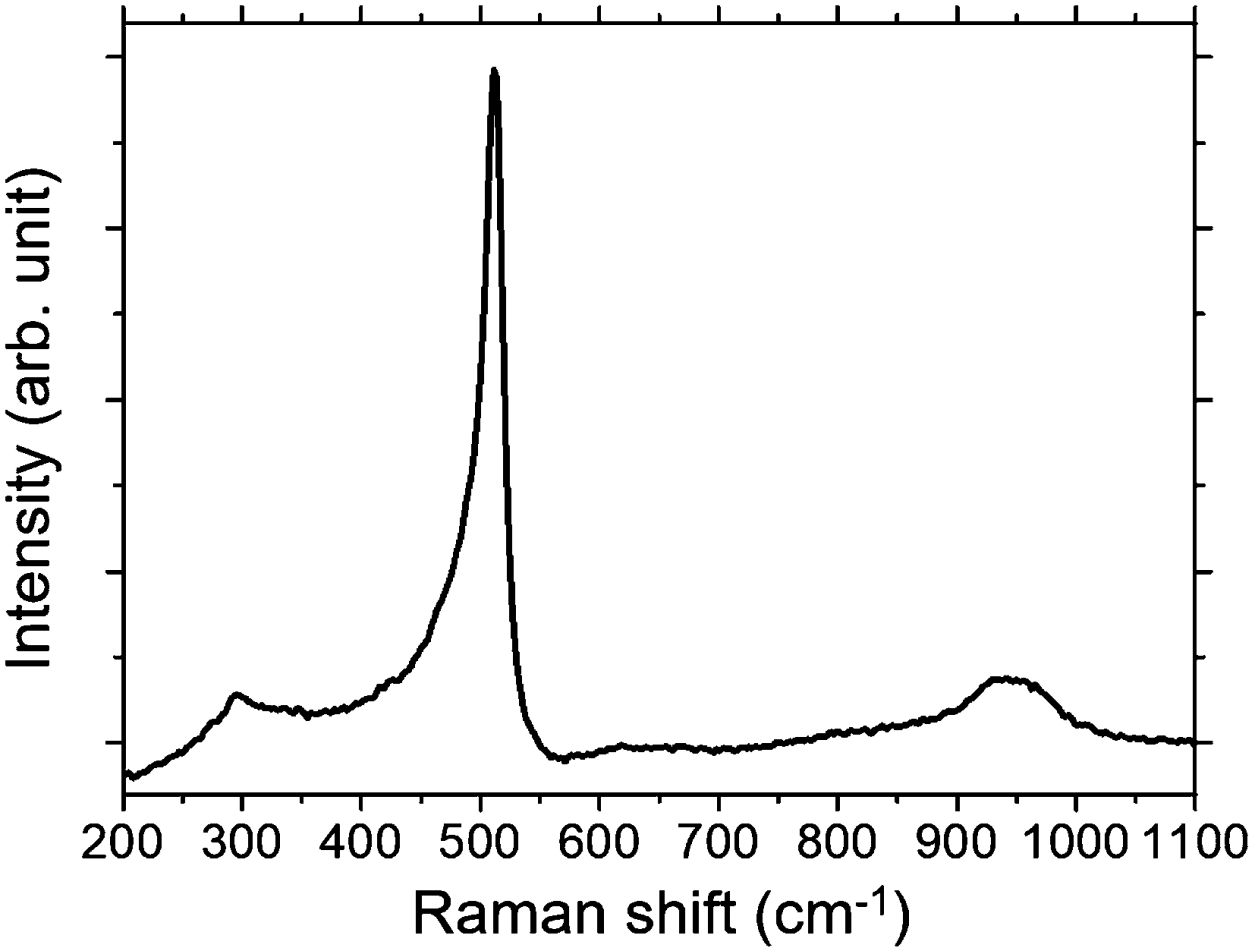
Raman spectra of the sample after 50 W of laser beam irradiation for 80 s.

The brown-colored area was widened as the irradiation time increased. After 80 s of irradiation with a 50 W power condition, most of the surface area of the mixture was brown, except for the crater area. The depth of the crater reached the bottom of the crucible. Figure 4 shows the Raman spectra of the sample. An enhanced peak of crystalline silicon was observed at 511 cm^−1^. The peaks at 295 and 941 cm^−1^ can be assigned to the second-order acoustic phonon mode and the second-order transverse optical phonon in crystalline silicon^24,25^. In addition, a higher power of laser beam radiation yielded the expected results. Clear peaks of crystalline silicon in the Raman spectra were observed for the 100 W irradiated samples; however, the diameter of the crater was increased, and the depth of the crater reached the bottom of the crucible after less than 40 s of irradiation. It is concluded that a power higher than 75 W is not desirable to obtain intact silicon in our experiments because the brown silicon band was damaged due to vigorous chemical reactions under high-power irradiation. The detailed experimental results regarding the process parameters, including the laser power, radiation time and molar ratio of the mixture, can be found in the Supplementary Information.

## Conclusion

In this report, a CO_2_ laser beam has been utilized as a heat source in the carbothermal reduction of silica to obtain silicon for the first time. Crystalline silicon was confirmed from the mixture of silica and carbon black under laser beam irradiation within a few seconds, as supported by the Raman spectra. We found that the laser beam could supply heat energy in the form of intensified heat flux to the mixture of silica and carbon black at an intensity above ~ 4.29 × 10^6^ W/m^2^ for carbothermal reduction. The intensity of the laser beam and N_2_ gas flow during the process were critical to obtaining silicon, and an increase in the intensity of the peaks of crystalline silicon was observed in the Raman spectra upon extending the radiation time. The beneficial aspects of the CO_2_ laser beam as a heat source in terms of feasibility were explored for the carbothermal reduction process for the first time. Although quantitative analysis of the conversion yield of silicon from silica is still in question at this time, we believe that our findings support one new method for extracting silicon from silica and carbon materials and may become the starting point for further process optimization and other silicon applications in the future.

## Supplementary information

Supplementary information 1.

Supplementary information 2.

Supplementary information 3.

Supplementary information 4.

Supplementary information 5.
