# Supplementary material for: Ultrafast carbothermal reduction of silica to silicon using a CO2 laser beam
Source: Sci Rep. 2020 Dec 10;10:21730. doi: 10.1038/s41598-020-78562-1 (PMC7729951; doi:10.1038/s41598-020-78562-1)
Supplement: Supplementary file 1 — Supplementary information 1. [file 41598_2020_78562_MOESM1_ESM.docx]

**Supplementary information**

**Ultrafast carbothermal reduction of silica to silicon using a CO_2_ laser beam**

# Seok-Ho Maeng1, Hakju Lee1, Min Soo Park1, Suhyun Park1, Jaeki Jeong2, Seongbeom Kim1,*

1Department of Mechanical Design Engineering, Kangwon National University, Samcheok-si, 25913, Republic of Korea

2Laboratory of Photomolecular Science, Institute of Chemical Sciences Engineering, Ecole Polytechnique Federale de Lausanne (EPFL), 1015 Lausanne, Switzerland

*sbkim81@kangwon.ac.kr

# Temperature measurements on the surface under the laser beam radiation and recorded videos

The temperature was measured using an infrared pyrometer, Model: IS 6-TV(MB30), LumaSense Technology, Inc. The pyrometer is capable of measuring temperature range from 600 to 3000 °C and also is capable of recording a video with a built-in CCD sensor. The focal point is adjusted to the measured spot to be 0.6 mm and the pyrometer was aligned to the center of the measured spot. The measurements were conducted in the air because it was not allowed to place any optics between the pyrometer and the measuring surface. Please see the separately attached video files.

25 W radiation video: video_25W.mp4

50 W radiation video: video_50W.mp4

27 W radiation video: video_75W.mp4

100 W radiation video: video_100W.mp4

(A date and time shown in the upper left side of the videos should be ignored because it displayed factory default values of the measurement software.)


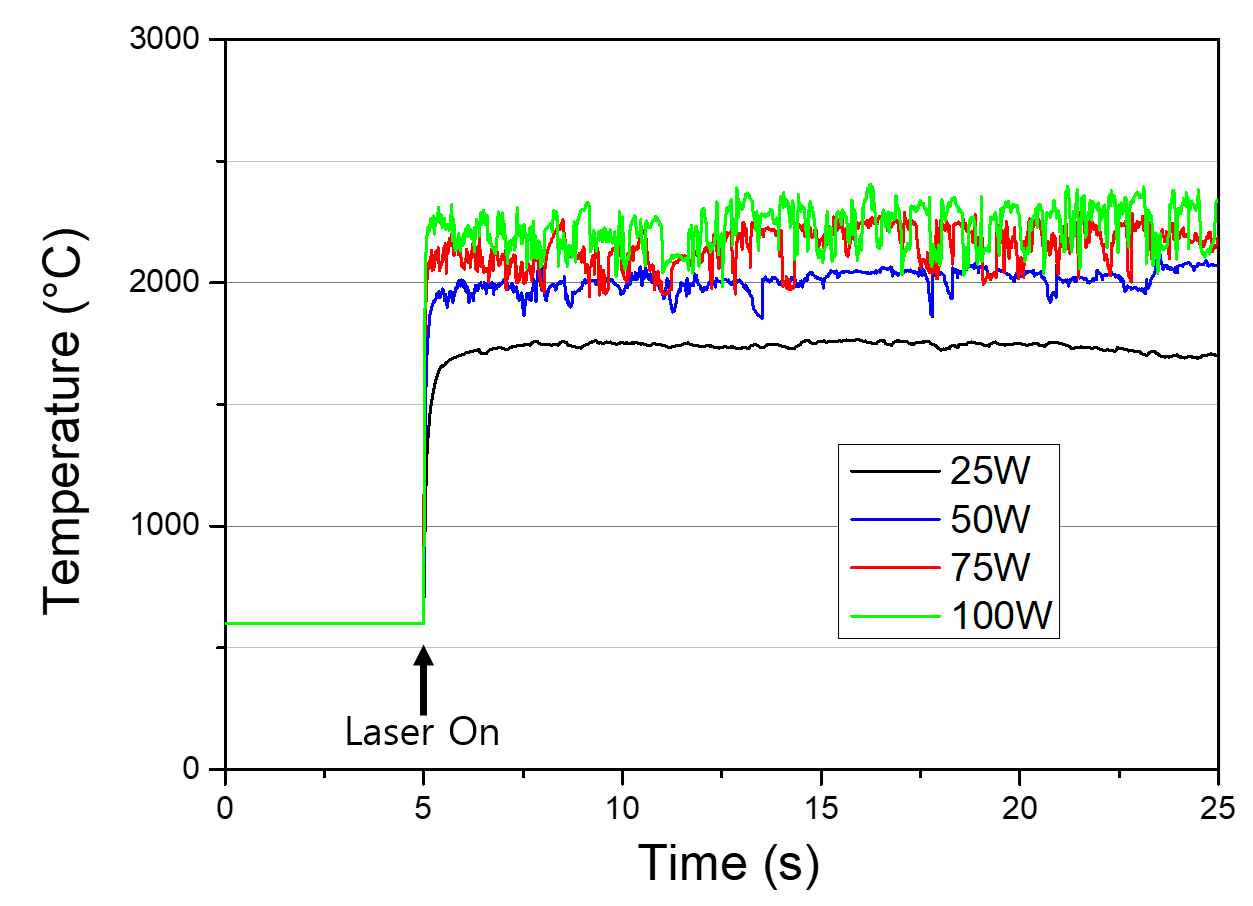


**Figure S1**. Temperature data

# Measurements of Raman spectrum

The Raman spectra were measured using Model: DXR3xi, Thermo Fisher Scientific Inc. The measurement conditions were 532 nm wavelength laser beam with 2.0 mW intensity, 10 Hz sampling frequency and the aperture with a 50 µm confocal pinhole was used.

# Digital images

A USB digital microscope was used for the digital images of the samples. To show an overall image of the mixture contained in a crucible, several digital images were assembled as shown in Figure S2. Therefore, the corners of the figures in the manuscript show blank spaces and the boundaries of each image may be seen as broken lines.


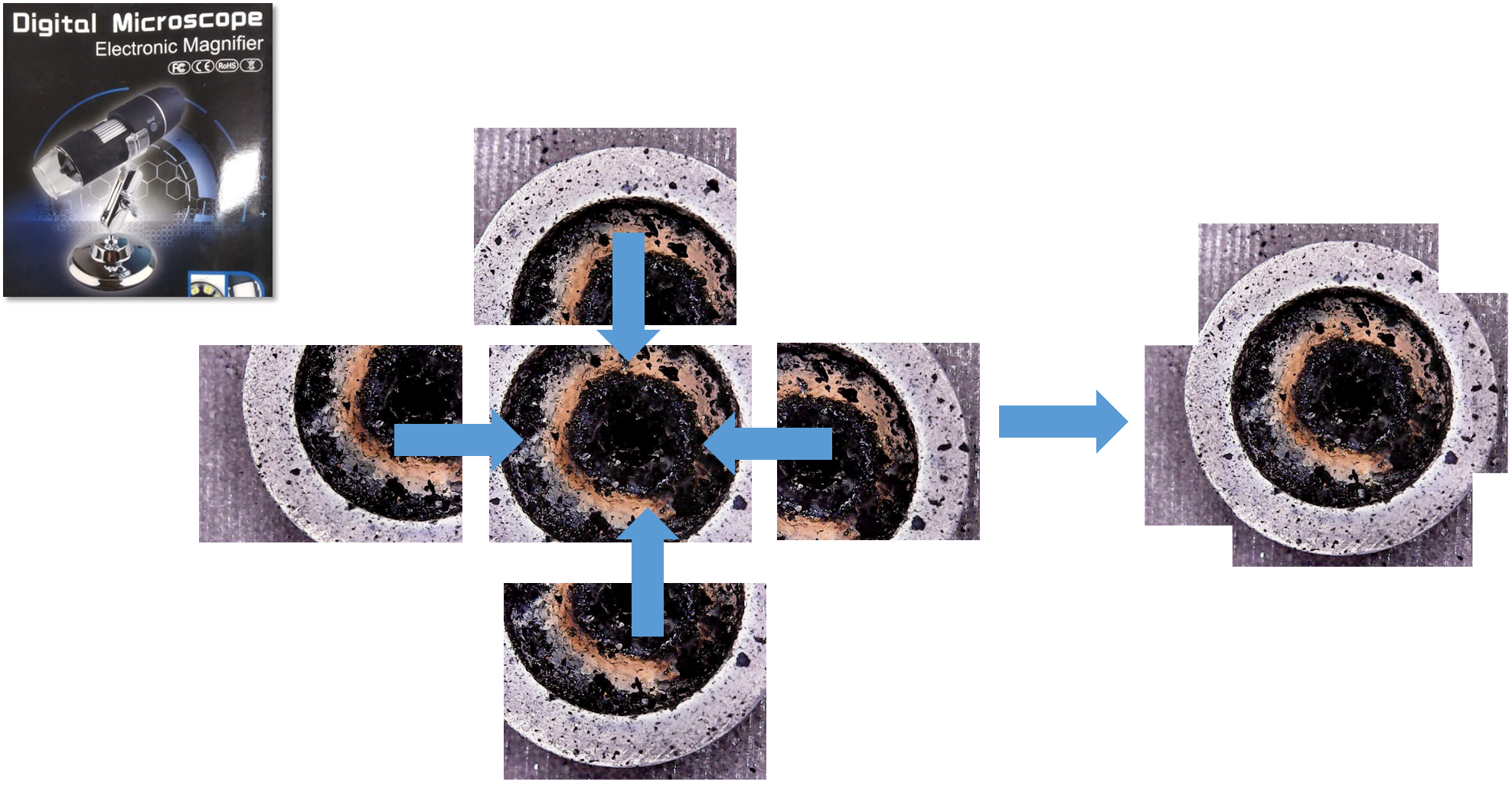


**Figure S2**. Assembling of the digital images


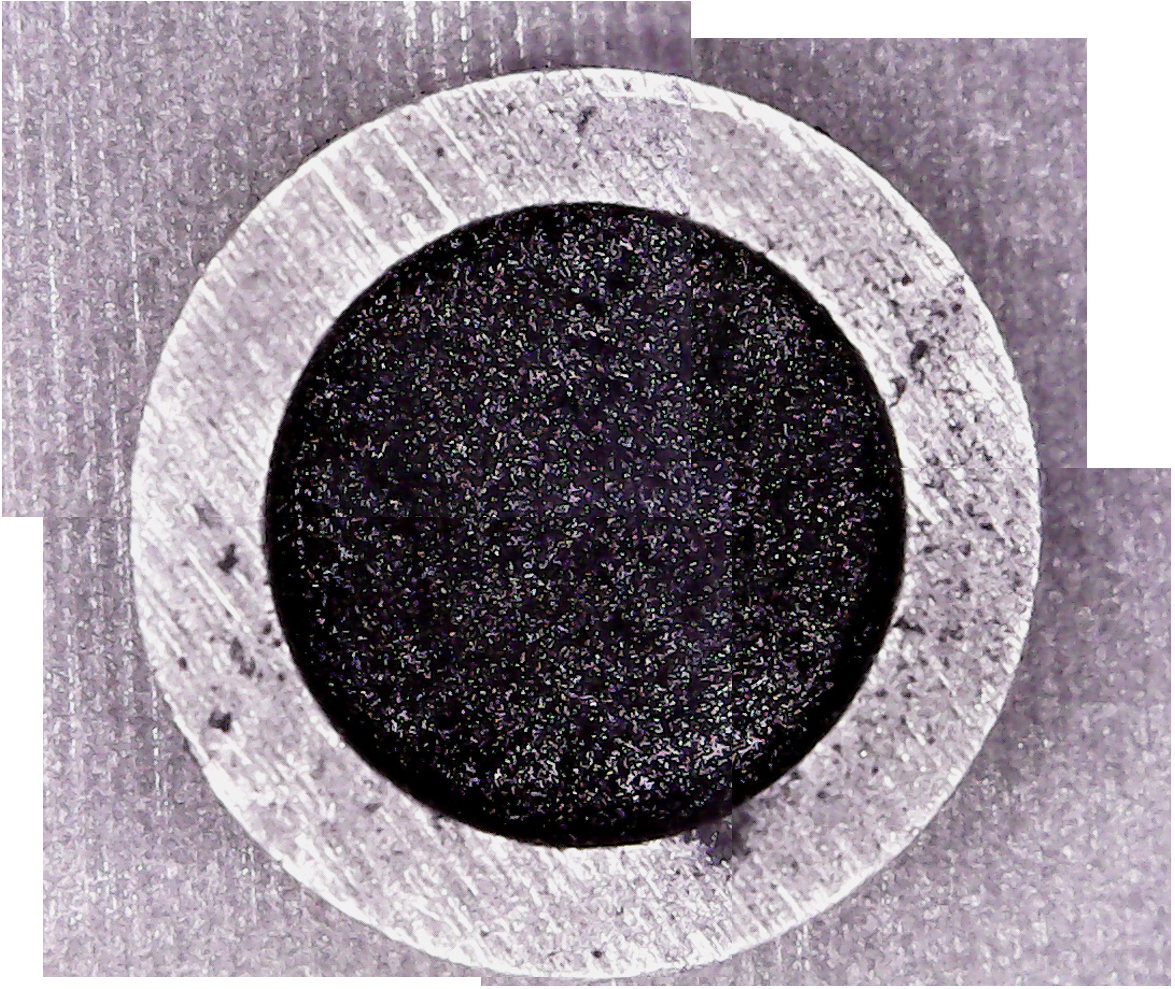


**Figure S3**. A digital image of the pristine mixture before the laser beam radiation

# Simulation of the laser beam intensity

TracePro, Lambda Research Corp. was used to simulate the laser beam intensity. 748501 incident rays were generated using a Gaussian profile source.

| Laser power 25W |
| --- |
| 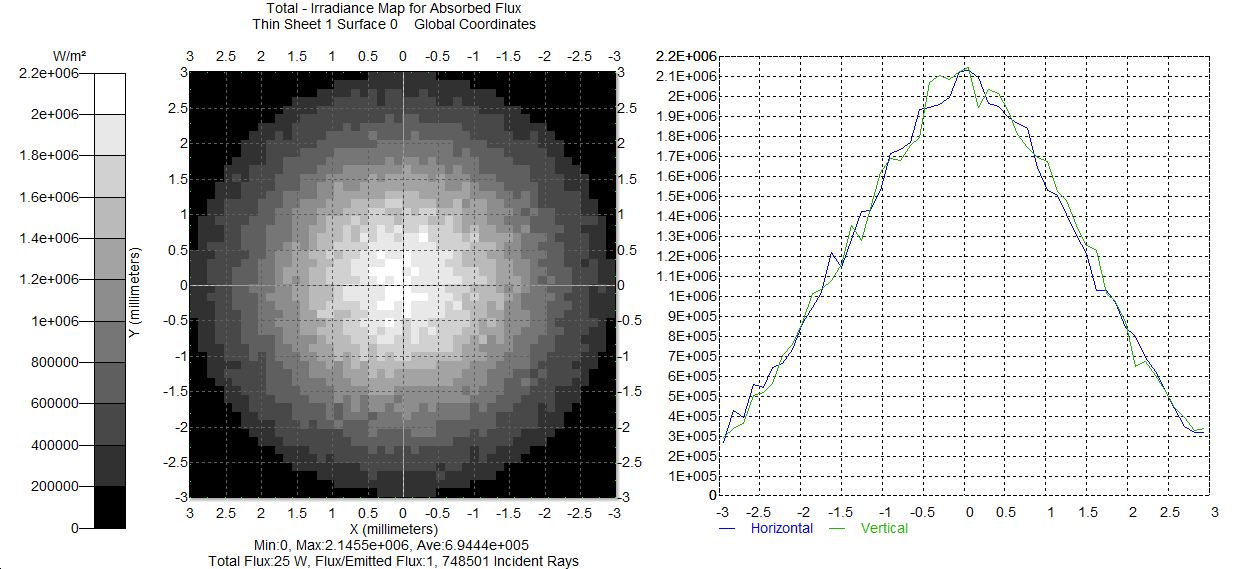 |
| Laser power 50W |
| 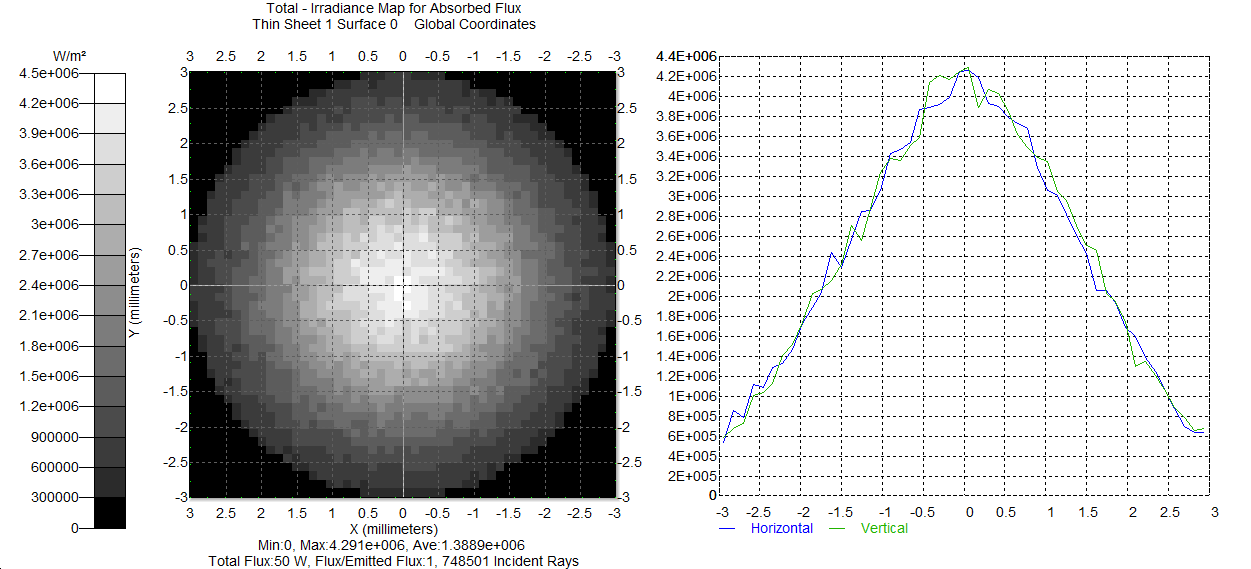 |

**Figure S4**. Laser beam intensity simulation

# Detailed experimental results regarding to the process parameters

By the design of experiments (DOE) tool, 64 sets of experiments were planned with a compromised number of experiments including 4 levels of each 4 process parameter. To approximate quantitative analysis of the results, the samples after the radiation were evaluated by the visual inspection qualitatively. Each digital image in Figure S5(1-3) corresponds with the condition in Table S1 by the number.

**Table S1**. Experimental conditions

| Experiment No. | Molar ratio of SiO_2_:C (1:X) | Pressure  (Torr, Controlled by N_2_) | Radiation time (s) | Laser total power (W) |
| --- | --- | --- | --- | --- |
| 1 | 1 | 50 | 10 | 100 |
| 2 | 1 | 50 | 20 | 75 |
| 3 | 1 | 50 | 40 | 50 |
| 4 | 1 | 50 | 80 | 100 |
| 5 | 1 | 100 | 10 | 25 |
| 6 | 1 | 100 | 40 | 25 |
| 7 | 1 | 100 | 80 | 50 |
| 8 | 1 | 100 | 80 | 75 |
| 9 | 1 | 200 | 10 | 50 |
| 10 | 1 | 200 | 20 | 75 |
| 11 | 1 | 200 | 40 | 25 |
| 12 | 1 | 200 | 80 | 100 |
| 13 | 1 | 400 | 10 | 100 |
| 14 | 1 | 400 | 20 | 25 |
| 15 | 1 | 400 | 20 | 50 |
| 16 | 1 | 400 | 40 | 75 |
| 17 | 2 | 50 | 10 | 75 |
| 18 | 2 | 50 | 40 | 25 |
| 19 | 2 | 50 | 40 | 25 |
| 20 | 2 | 50 | 80 | 50 |
| 21 | 2 | 100 | 10 | 50 |
| 22 | 2 | 100 | 20 | 75 |
| 23 | 2 | 100 | 20 | 100 |
| 24 | 2 | 100 | 40 | 75 |
| 25 | 2 | 200 | 20 | 50 |
| 26 | 2 | 200 | 20 | 100 |
| 27 | 2 | 200 | 40 | 50 |
| 28 | 2 | 200 | 80 | 25 |
| 29 | 2 | 400 | 10 | 25 |
| 30 | 2 | 400 | 10 | 100 |
| 31 | 2 | 400 | 80 | 75 |
| 32 | 2 | 400 | 80 | 100 |
| 33 | 3 | 50 | 10 | 25 |
| 34 | 3 | 50 | 20 | 75 |
| 35 | 3 | 50 | 40 | 100 |
| 36 | 3 | 50 | 80 | 50 |
| 37 | 3 | 100 | 10 | 25 |
| 38 | 3 | 100 | 20 | 50 |
| 39 | 3 | 100 | 40 | 100 |
| 40 | 3 | 100 | 80 | 100 |
| 41 | 3 | 200 | 10 | 50 |
| 42 | 3 | 200 | 20 | 100 |
| 43 | 3 | 200 | 40 | 75 |
| 44 | 3 | 200 | 80 | 25 |
| 45 | 3 | 400 | 10 | 75 |
| 46 | 3 | 400 | 20 | 25 |
| 47 | 3 | 400 | 40 | 50 |
| 48 | 3 | 400 | 80 | 75 |
| 49 | 4 | 50 | 10 | 75 |
| 50 | 4 | 50 | 20 | 25 |
| 50 | 4 | 50 | 20 | 25 |
| 51 | 4 | 50 | 20 | 100 |
| 52 | 4 | 50 | 80 | 50 |
| 53 | 4 | 100 | 10 | 50 |
| 54 | 4 | 100 | 20 | 25 |
| 55 | 4 | 100 | 40 | 100 |
| 56 | 4 | 100 | 80 | 75 |
| 57 | 4 | 200 | 10 | 75 |
| 58 | 4 | 200 | 10 | 100 |
| 59 | 4 | 200 | 40 | 75 |
| 60 | 4 | 200 | 80 | 25 |
| 61 | 4 | 400 | 20 | 50 |
| 62 | 4 | 400 | 40 | 50 |
| 63 | 4 | 400 | 40 | 100 |


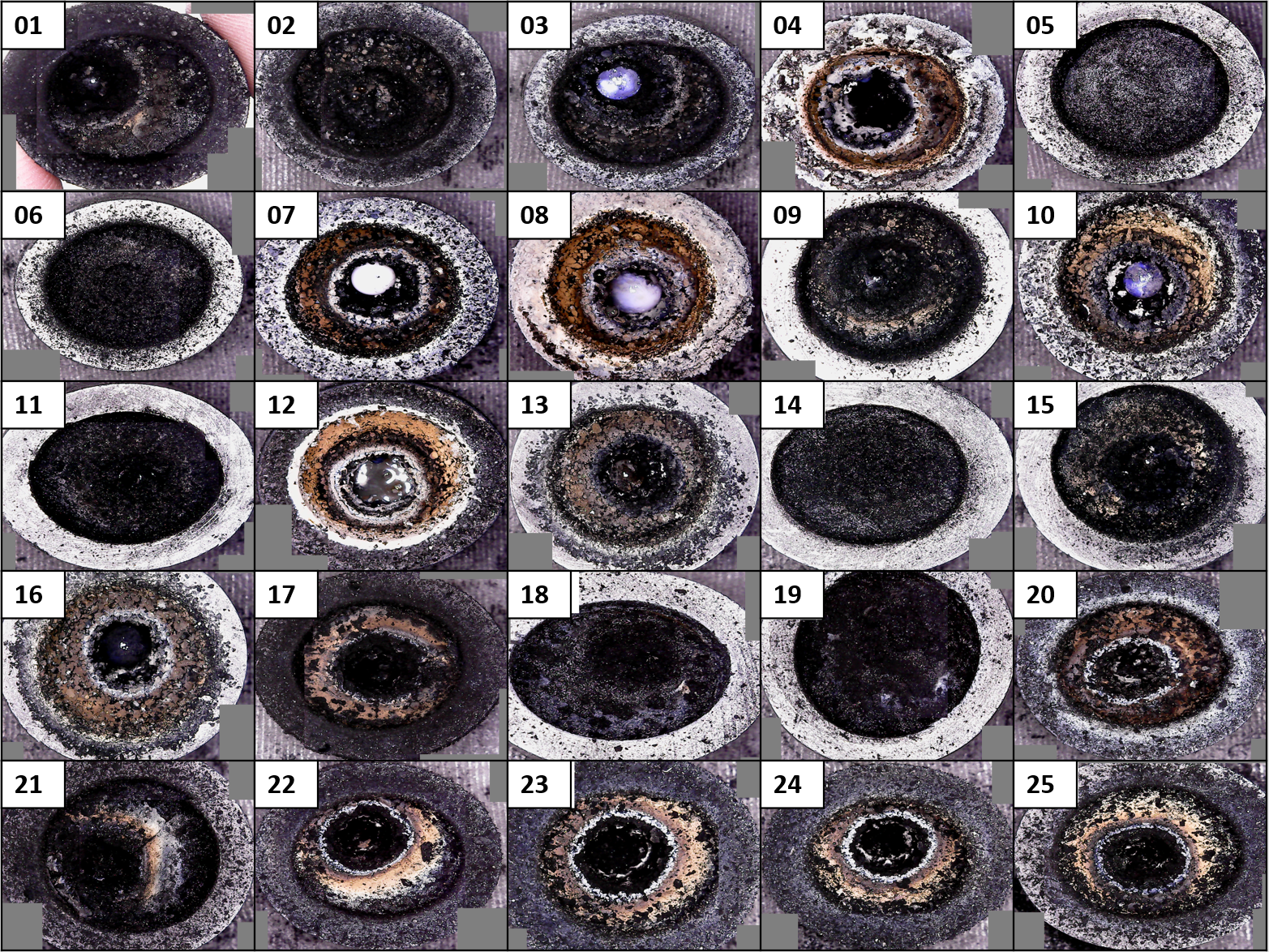


**Figure S5-1**. Digital images of the samples after the laser beam radiation


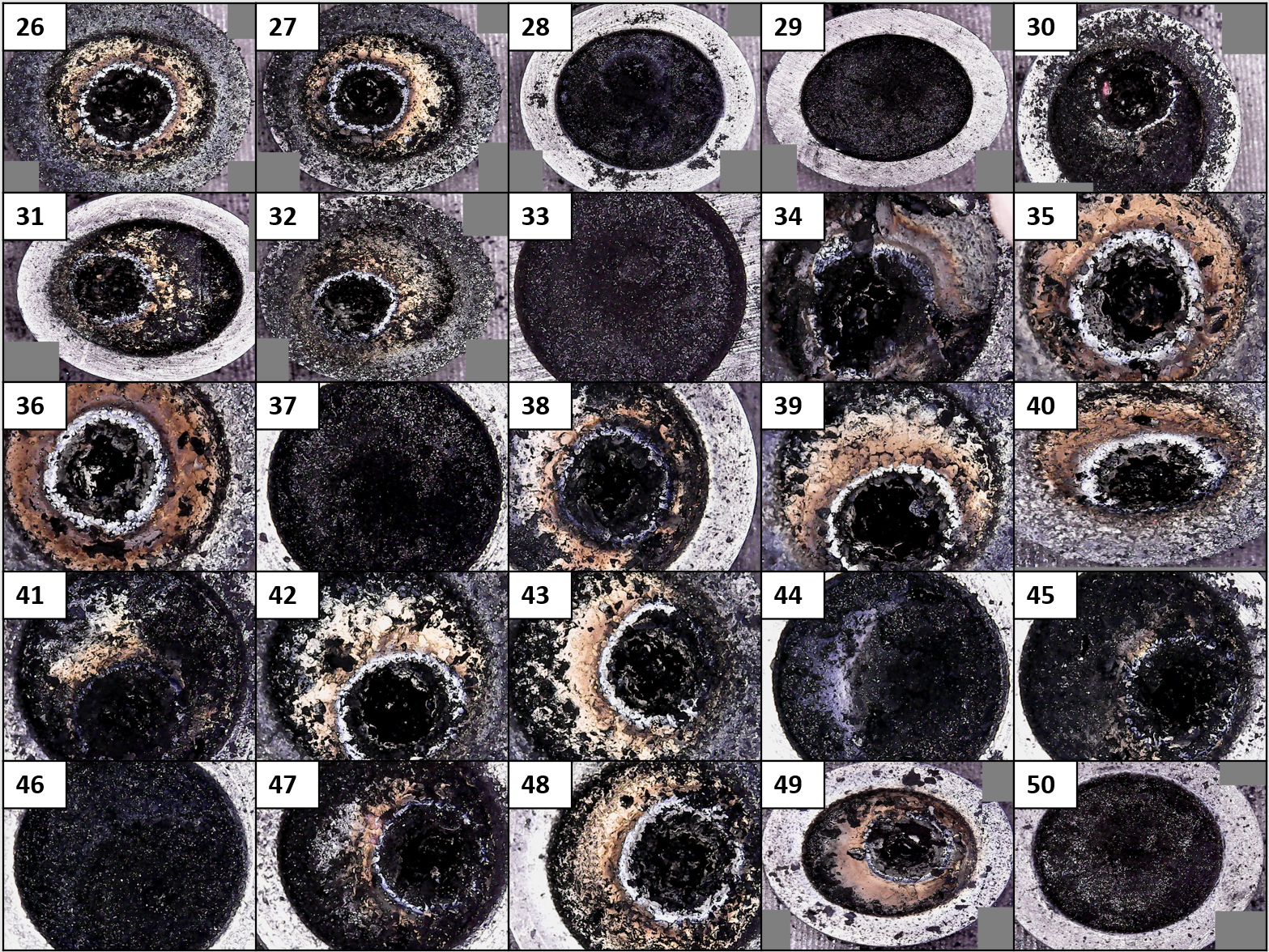


**Figure S5-2**. Digital images of the samples after the laser beam radiation


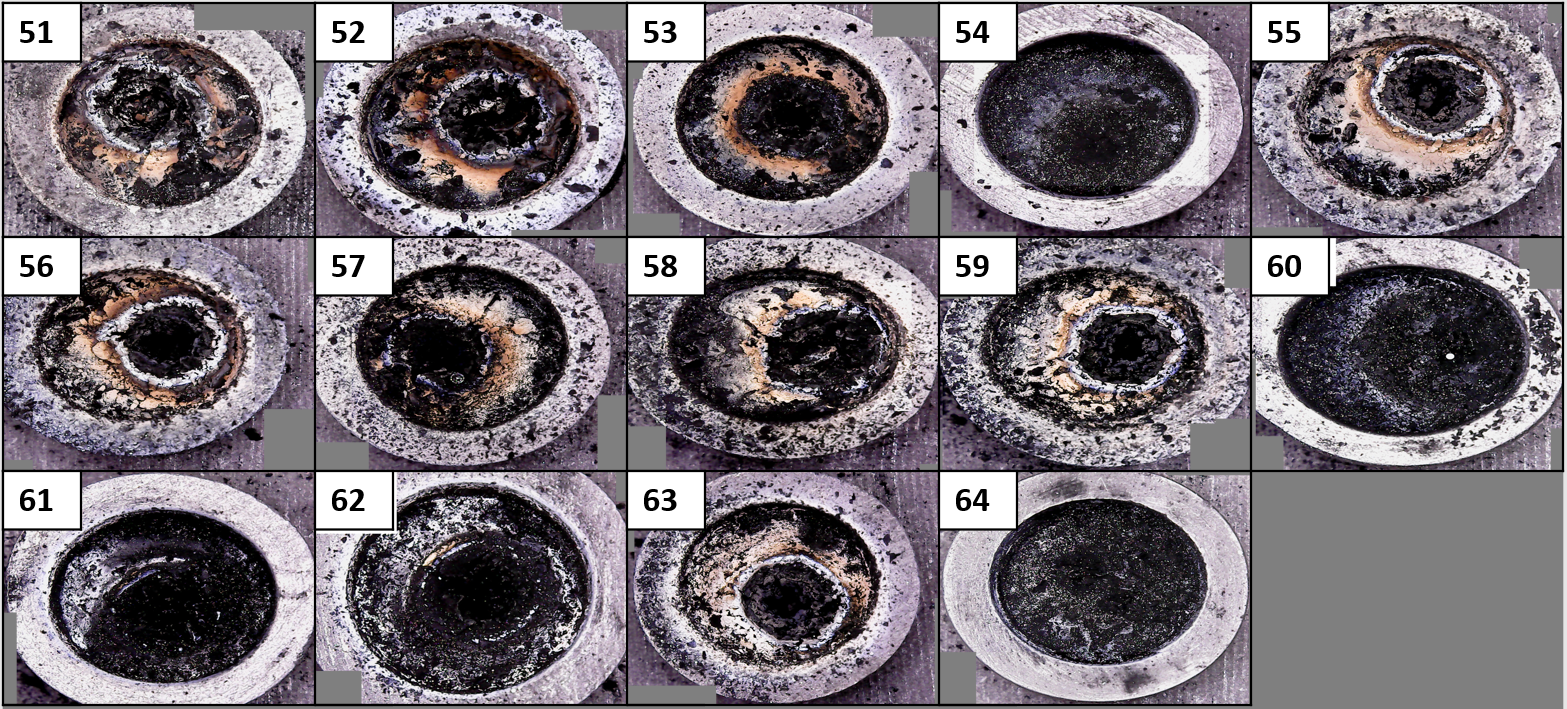


**Figure S5-3**. Digital images of the samples after the laser beam radiation

# X-ray diffraction of the irradiated sample

X-ray diffraction of the irradiated sample was measured using a model D/max-2500V, Rigaku corp. A small amount of powder on the brown color area was sandwiched between Kapton tape to seal the powder sample. All the sample preparation was conducted inside the N_2_ filled glove box to protect the sample from air exposure. The sealed sample was taken out of the glove box, then it was loaded on the XRD sample holder. Small peaks of cubic SiC are seen in Figure S6, however, that of the XRD peaks by silicon crystal is not detected.


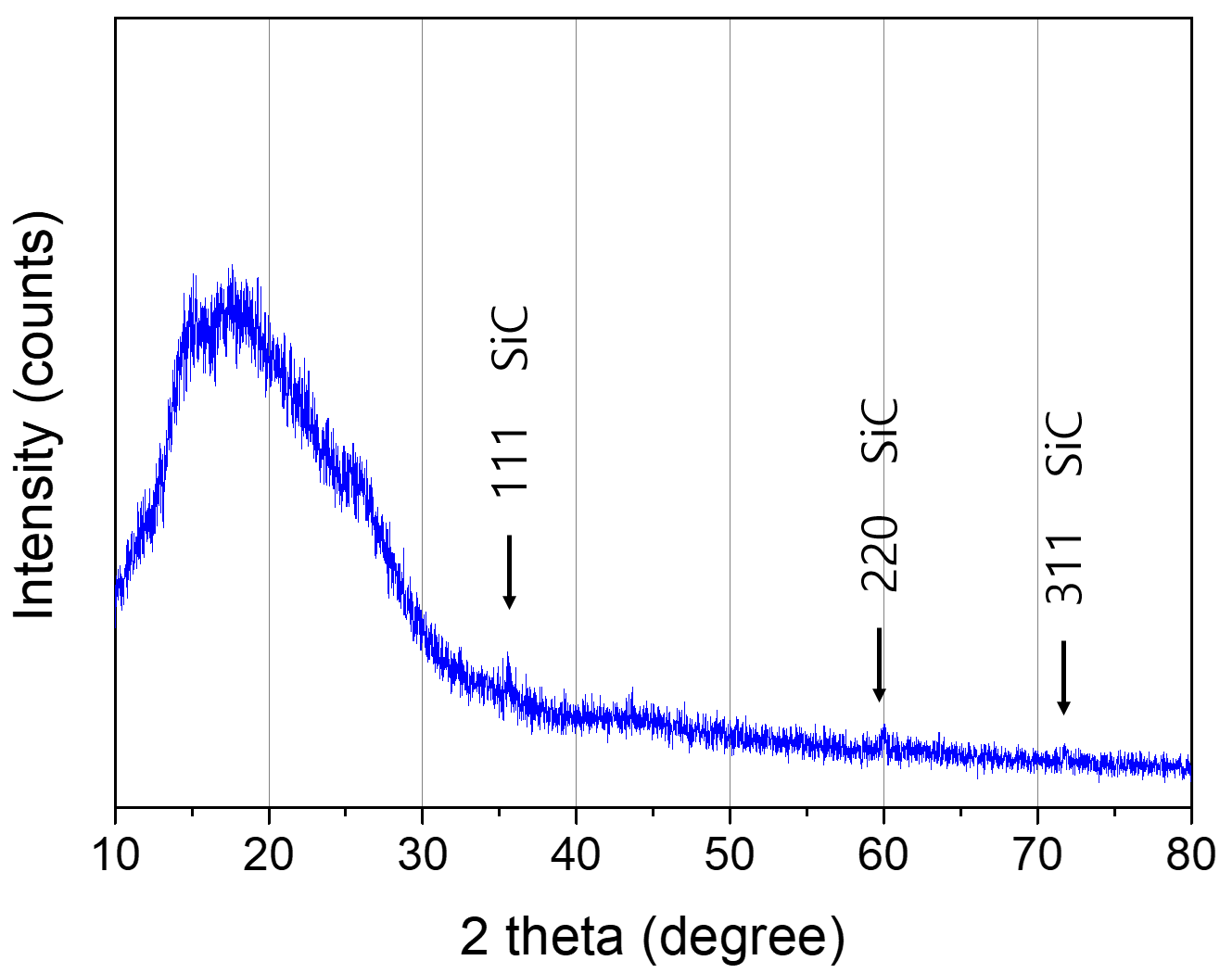


**Figure S6**. XRD data of the laser beam irradiated sample
